# Intestinal microbiota, probiotics and mental health: from Metchnikoff to modern advances: Part I – autointoxication revisited

**DOI:** 10.1186/1757-4749-5-5

**Published:** 2013-03-18

**Authors:** Alison C Bested, Alan C Logan, Eva M Selhub

**Affiliations:** 1 Complex Chronic Diseases Program, BC Women's Hospital and Health Centre, B223A-4500 Oak Street, Vancouver, BC V6H 3N1, Canada; 2 CAMNR, 775 Blithedale Avenue, Suite 364, Mill Valley, CA 94941, USA; 3 Harvard Medical School and Massachusetts General Hospital, 40 Crescent St., Suite 201, Waltham, MA 02453, USA

**Keywords:** Intestinal microbiota, Autointoxication, Depression, Anxiety, Probiotics, Microbial ecology, Lipopolysaccharide endotoxin, Diet, Intestinal permeability

## Abstract

Mental health disorders, depression in particular, have been described as a global epidemic. Research suggests that a variety of lifestyle and environmental changes may be driving at least some portion of the increased prevalence. One area of flourishing research involves the relationship between the intestinal microbiota (as well as the related functional integrity of the gastrointestinal tract) and mental health. In order to appreciate the recent scientific gains in this area, and its potential future directions, it is critical to review the history of the topic. Probiotic administration (e.g. Lactobacillus) and fecal microbiota transfer for conditions associated with depression and anxiety is not a new concept. Here, in the first of a 3-part series, we begin by reviewing the origins of the contemporary research, providing a critical appraisal of what has become a revisionist history of the controversial term ‘autointoxication’. We argue that legitimate interests in the gut-brain-microbiota connection were obscured for decades by its association with a narrow historical legacy. Historical perspectives provide a very meaningful context to the current state of the contemporary research as outlined in parts II and III.

## Series introduction

The global mental health crisis and prevalence of depression is increasingly being viewed, at least to some degree, as a consequence of modernization. There are numerous suspect candidates to explain what has been described as an epidemic increase in mental health disorders. These include, but are not limited to, socio-economic changes, urbanicity, alterations in dietary habits, sedentary behavior, excessive screen-based information consumption, lack of adequate sunlight, erosion of real-world (off-line) social support, and an overall disconnect from nature [1-3]. Researchers are beginning to explore the ways in which these and other factors may combine to influence mental health in contemporary society.

One area of flourishing research involves the neuropsychological consequences of alterations to gut microbiota (formerly referred to as “flora” or “microflora”) in conjunction with modern stressors, and an urbanized, Western lifestyle [4]. Almost a decade has passed since members of our group broke a 70-year-old scientific taboo by constructing a framework indicating that probiotics might play a beneficial role in conditions of human fatigue and depressive disorders [5,6]. Broadly speaking, ours was certainly not a new theory; it was, rather, a scientifically refined revival of select assertions that had been made a century prior. At our time of revival, in the early 2000s, the contention that the intestinal microbiota and the microbial-influenced integrity of the intestinal lining are of relevance to mental health disorders was, if it were to be suggested at all, a notion of nostalgia. Suggesting that intentional microbial manipulation could positively influence mental health, at least within scientific writing, was inevitably linked to the early 20^th^ century, to a time when some within medicine had veered off a rational course in a relatively short-lived obsession with so-called ‘autointoxication’ and ‘intestinal toxemia’ [7-11]. During this period the colon was viewed as the central road to a limitless array of illnesses, and in particular, those in the mental realm. In the early 1900s, much was written by high profile physicians and scientists concerning the ways in which the contents of the colon, most notably its undesirable microbial residents, could potentially contribute to fatigue, melancholia and the neuroses. The terms autointoxication, intestinal stasis, and intestinal toxemia were often used interchangeably to describe a process whereby intestinally-derived toxins could influence systemic health. While some authorities advocated drastic measures as treatment – e.g. surgical removal of portions of the colon – others preferred *First, Do No Harm*, and suggested manipulation of the intestinal microbiota by oral consumption of specific species of lactic acid producing bacteria. The latter treatment offered opportunity for the development of commercial microbial products, various pills and dairy-based beverages, which would be subsequently positioned for mental outlook and mental vitality.

Viewed from the vantage point of the year 2000, autointoxication and its related connections between intestinal health, microbes and mental health, was largely relegated to a somewhat embarrassing pseudoscientific footnote in medical history. Our renewed scientific discussion of orally administered microorganisms for mental health, the first of its kind for 7 decades, was radical, even outlandish, in the sense that it was inevitably associated with this historical context. By the year 2000, shallow and revisionist references to autointoxication came to define it as simply an unfounded fear associated exclusively with the toxic consequences of chronic constipation. These historical reviews have served to largely showcase the pseudo-medical exploitation (e.g. invasive surgeries, absurd “colonic irrigations”, “colon cleansers”, etc.) of intestinal toxemia’s core theoretical tenet – i.e. that intestinal microbes, directly and/or indirectly, could influence systemic health. Beneath this retrospective superficiality there was, in fact, a far more complex and nuanced discussion that had taken place between clinicians and scientists in the first third of the 20^th^ century. Many of these discussions, buried for too long beneath the modern focus on charlatanism and unnecessary surgeries, were indeed establishing an overlooked framework, the underpinnings of what we now refer to as the gut-brain-microbiome relationship. One such rational discussion was the 1930 ‘unifying theory’ put forth by dermatologists John Stokes and Donald Pillsbury [12]; although published in the *Archives of Dermatology*, it was a paper that was largely ignored and virtually unreferenced for 80 years. Excavated by Bowe and Logan, it has recently been the subject of a detailed review in *Gut Pathogens* journal [13]. The reader is referred to the review for more detail. Briefly, Stokes and Pillsbury provided an elegant theory whereby gastrointestinal mechanisms (including alterations to intestinal microbiota and intestinal permeability) could account for some of the overlap between emotional disorders and inflammatory skin conditions. Although Stokes and Pillsbury did not write on autointoxication *per se*, they published their theory at a time when the odds were against it being taken seriously. The publication of such ideas was far too close to autointoxication, and too far removed from any convincing science that was available at the time. As time passed, the theory would seem even more outlandish. To illustrate the point, consider that in 2002, at an annual dermatology meeting Stokes’ teachings were described as nonsense that could simply be ‘*swept into the dustbin of history*’ [14].

Here, in a three-part review, the authors will begin by examining both the absurdities and some of the more rational discussions and contentions made within the autointoxication period. This will provide a historical perspective and a point of reference for the remainder of the review - the contemporary gut-microbiome-brain research in mental health. To be clear at the very outset, it is not the purpose of this review to suggest that autointoxication as an umbrella term was universally “correct”, or to deny that it was, and indeed remains today, the terrain of charlatanism, shysters and pseudoscience. It is not our desire to reestablish autointoxication as a legitimate descriptive term for use within contemporary medical lexicon; and obviously, your present authors are under no illusion that the current mental health crisis is mediated by want for psyllium husks or high colonics. Rather, our contention, in the context of the advances to be discussed in Parts II and III, is quite simple – modern scientific technique is demonstrating that there is more to the legacy of intestinal toxemia than that which states it was all nonsense.

### Part I

#### Autointoxication revisited

‘*The control of man’s diet is readily accomplished, but mastery over his intestinal bacterial flora is not…the innumerable examples of autointoxication that one sees in his daily walks in life is proof thereof. They are the cases that present…malaise, total lack of ambition so that every effort in life is a burden, mental depression often bordering upon melancholia, frequent attacks of indefinite abdominal pains due to flatulency, sudden attacks of acute diarrhea alternating with periods of constipation…A battle royal must be fought and when this first great struggle ends in victory for the Bacillus bulgaricus it must be kept on the field of battle forever at guard…*’

#### Bond Stow, M.D., on autointoxication and *Lactobacillus bulgaricus* – Medical Record Journal of Medicine and Surgery, 1914

From the quote above [15], drawn from a mainstream medical journal of the day, one can see the unbridled enthusiasm with which some physicians embraced the link between autointoxication, mental vitality and intestinal microbes. The keynote symptoms of autointoxication were primarily digestive, most notably (as described in the quote above) alternating constipation and diarrhea. Experts at the time made it clear that constipation is not necessarily coincident with autointoxication, and that it may be even more likely to occur in those with melancholia and more fluid-like or semi-solid stools [16].

In the 1860s, German physician Hermann Senator raised the notion that systemic disease, including mental health disorders, could be rooted in intestinal ‘self-infective’ processes [17]. Scientists had begun to experiment with aqueous extracts of putrid or decomposing meat, fish and dairy products, and when small amounts of these isolated chemicals were introduced into systemic circulation of various animals, the results were often fatal. In 1887 the French physician Charles Bouchard published his famous *Lectures on Autointoxication in Disease* and the theory of autointoxication gained international recognition. The premise was fairly simple; the colon was, as Bouchard put it, ‘*a receptacle and laboratory of poisons*’, the chemical breakdown products of bacterial action on food material (putrefaction) could be absorbed systemically, and if not handled properly the end result could manifest as increased susceptibility to disease over time. In the preface to the English translation (1897) of Bouchard’s *Lectures*, physician Thomas Oliver stated that role of harmful intestinal bacterial-derived chemicals in mental diseases was part of the new frontier of autointoxication research [18], and that ‘*man is constantly standing, as it were, on the brink of a precipice; he is continually on the threshold of disease. Every moment of his life he runs the risk of being overpowered by poison generated within his system*’. Proponents contended that autointoxication was largely a chronic process that could be enabled by lack of adequate stomach (hydrochloric) acid production, inflammation of the intestinal wall, other disease burdens such as influenza, and importantly, nervous excitement. Notably, military physicians [19] suggested that ‘*circumstances may arise in the stress of war when the soldier may be peculiarly susceptible*.’ In other words, stress could set the stage for autointoxication, which in turn, would promote a more rapid deterioration in mental health.

In general, this was not a tough sell to medical professionals of the time - the germ theory of disease promoted by Louis Pasteur and others was a scientifically sound construct in which to understand and replicate the mechanisms whereby specific microbes initiate certain diseases. The broad notion that fecal matter contains harmful poisons is as old as recorded history, Greek historian Herodotus [20] reported that the ancient Egyptians used enemas three days per month along the lunar cycle because they are ‘*convinced that all the diseases incident to man have their origin in the food that he takes*’. Enter the rudimentary scientific reports of the late 1800s indicating that certain food breakdown chemicals were potentially fatal, at least in animals, and the theory was primed for popularity. In 1898, physician Daniel R. Brower of Rush Medical College published one of the first original papers on autointoxication and melancholia in the *Journal of the American Medical Association* (JAMA). Brower suggested that lack of stomach acid might play a role in promoting microbial growth in the intestines and a subsequent higher production of toxic products. In addition to indole, skatole and other toxic products, he suggested that intestinally-derived lactic acid may be at play, an area of research we will discuss in Part II. He acknowledged that under normal circumstances that intestinally-derived toxins are easily handled by the liver and kidneys, his concern was how these detoxification pathways might be overrun in melancholia [21]. Brower did not dispute the purely psychological aspects of depression associated with grief, loss, and other major life changes; however, he felt strongly that increasing rates of melancholia in urban and Western nations were a by-product of civilization, and some of that increasing risk might be mediated by changing dietary habits and potential toxins arising from the gastrointestinal tract [22].

Others papers would follow [23], and by 1905, there was a growing acceptance of autointoxication among some ranks of psychiatrists, mental hospital superintendants, and other physicians. Concerning melancholia, many agreed there was often at least some degree of involvement related to autointoxication originating from the gastrointestinal tract. The problem, of course, lamented one physician, was that ‘*unfortunately the only criterion we have for judging of the existence of autointoxication is the therapeutic result*’ [24]. The toxins in question remained obscure – i.e. were these toxins the by-products of bacterial action on food residue, where they direct secretions from the bacteria, or even portions of the bacterial structure? The editors of the *Albany Medical Annals* also stated in 1905 that ‘*much attention has been given in the last few years to the relations of mental disturbance with autointoxication, and the tendency has been toward a liberal interpretation of the latter condition as an etiological factor*’ [25].

Autointoxication as a condition and/or an etiological factor was vague, thus allowing for liberal use and an easy default explanation for incredibly complex disorders such as depression. In the years following, the contributions of famed British surgeon Sir Arbuthnot Lane and Nobel-Prize-winning microbiologist Ilya (Elie) Metchnikoff would make autointoxication and even more tempting explanation for mental health disorders. Since their work gained the attention of international print media, a period of popular and commercial acclaim to the autointoxication hypothesis would begin in earnest.

#### Sir Arbuthnot Lane

Much like Bouchard, Lane also viewed the colon as a simple ‘sewage system’, however, he focused his attention on the internal suspension points of the bowel, which in conjunction with gravity and altered dietary habits subsequent to the industrial revolution, set the stage for sections of colonic stagnation. Within this ‘cesspool’, as Lane called it, the normal intestinal bacterial flora was said to be altered and a migration of bacteria toward the small intestine would encourage an even greater absorption of intestinal toxins. The symptoms, as usual, were far and wide, although they were most notable in the gastrointestinal and mental realms – dyspepsia, abdominal pain, constipation alternating with bouts of diarrhea, malaise, melancholia, incapacity for prolonged mental or physical exertion, insomnia and neuroses [26-28]. The catch-all diagnosis for all of these symptoms - a cluster of modern day co-morbid irritable bowel syndrome, chronic fatigue, myalgic encephalomyelitis, fibromyalgia, anxiety and depressive disorders - was neurasthenia, and Lane was convinced that neurasthenia was almost always a matter of colonic toxemia. Beyond lifestyle interventions and internal disinfectants for mild or early-stage colonic stasis, a chronic state, in Lane’s view, could only be resolved by surgery, typically a colectomy or complete colon bypass (so-called short circuit). Claims of surgical cures abounded; in a short period of time colectomy was being recommended, as reported by a physician of the time, for ‘*comparatively trivial symptoms*’ [29]. Never mind that even with the best of surgical skill and technique, the mortality rate was over 16%, patients commonly returned for follow-up surgeries, enduring worse pain and suffering vs. the pre-surgery state, and even for cases of clinical ‘success’, the recovery period was in the range of two years [29].

Lane’s claims of success were a stimulus for yet more surgeries to ‘cure’ mental health disorders under the autointoxication-related umbrella term of ‘focal infections’. Once again, invasive procedures were justified by theory. Focal infections were translated as localized infections, most notably chronic bacterial infections of the periodontal tissue and/or the colon. These infections, ranging from the visibly obvious to the microscopically subtle, were thought by some to be a driving force in the *causation* of mental illnesses (via a systemic toxin load that would influence nervous system function). Even among those who strongly suspected that chronic low-grade infective processes (e.g. oral sepsis) was associated with mental disorders, they often placed it as a risk factor in the context of heredity and an already overrun state (poverty, mental overwork, poor nutritional status). However, at least one New Jersey physician, Henry Cotton, believed focal infections to be the initiator of virtually all forms of psychoses, mood and behavioral disorders. The difference between Cotton and almost all of his contemporaries, those who might presume that endotoxin exposure and systemic low-grade inflammation could influence general health, was that he forged ahead with horribly invasive interventions based solely on his convictions. Under Cotton’s direction, thousands of teeth were extracted and hundreds of colectomy operations were performed in his State-run mental hospital, with a spectacular remission rate claimed to be as high as 80%. Lost in the details of recovery claims was a mortality rate of 30% among 250 patients subjected to colonic surgeries in just 3 recorded years (1919–1922), procedures initiated, to remind the reader, for the purpose of mental health [30]. Also lost in the recovery claims were the actual statistical details, the verifiable results backing up the startling success. Even as late as 1926, Cotton’s colleague, prominent New York surgeon John W. Draper, was writing up colectomy cases in the *Annals of Surgery*, claiming a mental illness recovery rate 2.3 times higher than that involving standard care [31].

Cotton and Draper continued on their quest despite evidence of its futility. In 1922–23 highly respected microbiologist Nicholas Kopeloff, along with future presidents of the American Psychiatric Association, Clarence O. Cheney (1935–36) and George H. Kirby (1933–34), set up what are likely to be the first controlled trials in the history of psychiatry. In separate studies involving 60 and 120 adults with more severe forms of mental illness (schizophrenia, manic-depression), the participants were divided into an oral surgery group (all tonsils removed, plus removal of an average of 5 teeth per subject) vs. half in a non-surgical standard care group [32]. Contrary to the claims of Cotton, the researchers were unable to find any differences in long-term recovery rates between the two groups, discrediting the claim that surgical removal of teeth and tonsils *alone* was responsible for recovery. Kopeloff and colleagues, while vehemently opposing the idea of pulling teeth and tonsillectomies as a cure, would not dismiss local infections as completely irrelevant to mental health. Perhaps they were right in that regard; modern research continues to show strong associations between microbial-induced periodontal disease, chronic low-grade inflammation, and higher risks of cardiovascular disease, diabetes, lung disease, and adverse pregnancy outcomes [33]. How this fits in with separate contemporary research on periodontal disease, psychological stress and depression [34], remains an open question. Nonetheless, Cotton’s cure – total or partial endentulism and denture wearing – is now known to be associated itself with systemic inflammation and depression [33,35,36]! Kopeloff would go on to write extensively about the alternatives to surgery, including the general intestinal value of *Lactobacillus acidophilus* – ‘*constipation, which is so prevalent, does much to aggravate infective conditions. The habitual use of cathartics is vicious, and a rational treatment such as that employing acidophilus, the twin brother of Bulgarian bacillus, is much more desirable*’ [37].

#### Elie Metchnikoff

Metchnikoff was in agreement with the most of the viewpoints of Lane; the two were reported to be well acquainted with one another [28]. However, as a microbiologist Metchnikoff would gain fame via his preliminary research indicating that orally consumed lactic acid bacteria could combat the dangers of autointoxication. Although he focused mainly on autointoxication in relation to aging, and specifically on the ability of *Lactobacillus bulgaricus* to slow arteriosclerosis and other objective markers of age-related decline, the consequence of unchecked intestinal toxin production was extended to neurasthenia and overall quality of life [38-40]. In a special contribution to *Cosmopolitan* (1912), Metchnikoff wrote “*In effect, we fight microbe with microbe…there seems hope that we shall in time be able to transform the entire intestinal flora from a harmful to an innocuous one…the beneficent effect of this transformation must be enormous*” [41].

Contemporaries of Metchnikoff built volumes of clinical claims on the basis of his hypotheses and preliminary work with rabbits, guinea pigs and monkeys. For example, Albert Abrams, a San Francisco physician, stated in his 1914 text entitled *The Blues, Causes and Cure*, that one of the keys to recovery from depressive symptoms was correction of autointoxication via the use of ‘*antagonistic microbes*’ and that ‘*unquestionably, the liquid Lactobacilline, as it is called, is the most efficient*’ [42]. Within the 1915 edition of *Therapeutics of Internal Diseases*, edited by Harvard medical doctor and the president of the Association of American Physicians, Frederick Forchheimer, it was stated that ‘*there are many varieties of lactic acid bacilli tablets on the market for direct ingestion or for the preparation of a lactic acid milk; but none of these have the efficiency or produce the palatability of milk which is common to the true Bulgaricus preparation known as liquid lactobacilline…the signs of autointoxication disappear slowly, and, therefore, for curative and hygienic purposes it is advisable to continue to the use of lactobacilline more or less continuously for several months*’ [43]. Chief among the reasons for the administration of lactobacilline and other interventions, it was further stated in the text that ‘*patients suffering from neurasthenia, melancholia, hypochondriasis, and allied conditions are much more sensitive to the toxic effects of intestinal putrefaction than are those that have a normal nervous system. There is here illustrated a vicious circle: that is, depressed conditions of the nervous system very readily lead to constipation and autointoxication; and the latter condition, in turn, aggravates and excites the nervous symptoms’*[43].

Lactobacilline (or Lacto-bacilline of Metchnikoff) was but one of a long list of *Lactobacillus* preparations that would be produced commercially for the treatment of autointoxication and its sequelae. The first wave of products focused on Metchnikoff’s Bacillus bulgaricus. Another example was Berlin Labs of New York and their Intesti-Fermin tablets positioned for increasing mental vitality and the treatment of neurasthenia. Berlin Labs secured full page advertisements in a wide variety of periodicals, stating that ‘*Metchnikoff’s great discoveries now procurable in tablet form*’ and that Intesti-Fermin ‘*promotes physical and mental health and provides a truly scientific aid to high efficiency in every-day life*’ [44]. Metchnikoff distanced himself from the commercial products and brought suit against Berlin Labs for using his name in the advertisements [45]. By 1917 the *Druggist’s Circular* listed some 30 different commercially available Bacillus bulgaricus preparations [46]. However, by the early 1920s L. bulgaricus declined in popularity and L. acidophilus was the species of commercial and clinical choice. After publications by Yale University scientists Leo F. Rettger and Harry A. Cheplin and colleagues [47], those showing that L. acidophilus can and does live and develop in the intestines of humans and animals - while L. bulgaricus does not - many of these same companies would completely abandon the L. bulgaricus in favor of L. acidophilus.

#### Acidophilus as a mental tonic

The early 1920s witnessed a second wave of enthusiasm for the use of oral ‘bacteriotherapy’ as a means to positively influence mental health. In North America various producers of acidophilus milk – e.g. Lederle Antitoxin Laboratories, Walker-Gordon Laboratories, Cheplin’s Biological Laboratories - began widespread distribution in tandem with marketing campaigns within medical and popular print media. During the 1920s it was commonplace to see competitive advertisements in medical journals for *Lactobacillus acidophilus* preparations that were “American Medical Association Council Accepted” [48]. Although reluctant to officially list such remedies in the absence of scientific support, the American Medical Association Council on Pharmacy and Chemistry did so at the time because their own nationwide survey of clinicians deemed the Lactobacillus preparations to be of some clinical value [49]. Within medical journals the advertizing was largely restricted to viability of the bacteria and general value. However, within popular press the claims were more direct – for example, Walker-Gordon acidophilus milk campaign in New York newspapers [50] stated “*The results, as thousands of physicians and users testify, are nothing short of amazing. Not only a banishing of mental and physical depression, but a flooding of new vitality throughout the system*”. Meanwhile, the competition, Lederle, claimed in the same New York newspapers that “*There can be no question of the efficacy of Lederle acidophilus milk…restoring your energy to its maximum*”, going on to urge consumers to choose Lederle over the competition [51]. In support, there were opinion-based newspaper editorials from various scientists claiming value of acidophilus therapy – “Acidophilus Milk Helps Avoid Run-Down State” was the headline produced in the syndicated column of one nutritional scientist [52].

In the absence of convincing scientific evidence, this pattern of market-driven assumptions (and consumption) carried on through the early 1930s. In one of its last campaigns of the genera, in 1932, Walker-Gordon placed advertisements in the New York Times under the banner “Keep a Cheerful Outlook” with the text following claiming that “*It’s a fact – your doctor will agree – that your attitude toward things in general is largely influenced by the condition of your intestinal tract*” [53]. They suggested a 30-day challenge of acidophilus milk consumption to “*see if you don’t feel better and brighter*”. Walker-Gordon also provided a complimentary copy of its booklet ‘Out of the Blues’, the acidophilus and mood story, to its consumers [54].

#### Scientific beginnings of the gut-brain-microbial connection

Beyond Lane, Metchnikoff and Madison Avenue marketing teams (Walker-Gordon’s campaigns were overseen by a medical doctor [55]), other scientists of the day were making preliminary, although not as well publicized, contributions to the field of intestinal ‘toxemia’ and mental health. For example, in 1906, pathologists David Orr and Richard Rows began to investigate the ways in which alimentary canal-derived microbes, including those involved in acute gastroenteritis, can gain access to the lymphatic channels and influence sympathetic nervous system function. Although they were focused on serious forms of nerve damage (e.g. tabes dorsalis), they acknowledged that they were motivated to investigate gut microbes because mental disorders, as they put it, are often of intestinal origin [56,57]. Around the same time, in 1904, Alice Johnson and Edwin Goodall reported that institutionalized adults with mental illness have a more pronounced blood reaction to colonic bacterium. Specifically, they found that in a sample of 82 adults with acute (defined as 1–6 months duration) melancholia or mania, 50% showed agglutination of serum when exposed to intestinal-derived Escherichia coli (E. coli). The control group agglutination reaction to E. coli was 15% [58]. This finding could be interpreted in a number of ways, one of which was that the intestinal mucosa may vary in its permeability to gut-derived organisms or parts thereof. Researchers from the State College of Washington reported that orally administered heat-killed E. coli would only cause a systemic agglutination reaction in some healthy rabbits and not others, suggesting that there may be differences in intestinal permeability [59].

In 1906, New York physician Fenton Turck reported on the ability of psychological stress to increase intestinal permeability to gut microbes in animals [60]. Through more than a decade of investigation he also showed that intestinal permeability could be induced by states of fatigue, various dietary components, and withdrawal of adequate blood supply to the intestines. In one study Turck demonstrated that the dietary addition of high-fat bread (fried in cottonseed oil for 30 minutes, presumably also forming significant trans-fatty acids) caused intestinal permeability vs. control diet without the fried bread. Turck found that animals fed a high-fat diet, in addition to the usual vegetable chow, showed an increase in bacterial translocation to tissues. Moreover, he reported that in rats and mice fed a diet inclusive of meat extractives (flavorizing components) there was a pronounced intestinal permeability to *E. coli* vs. standard diet [61,62]. Meanwhile, J. George Adami, a staunch critic of Lane’s colectomy solution, was pleading with the scientific community to take serious his contention that many of those with so-called autointoxication were actually experiencing symptoms of a low-grade immune response [63,64]. This immune response to systemic gut bacteria was subsequently linked, at least theoretically, to melancholia [65].

Turck was also convinced that the colon was a reservoir of endotoxins (including those produced by *E. coli*), and that once a threshold was passed in specific clinical cases, the endotoxins can gain systemic access, ultimately aiding in the promotion of disease states [66,67]. Although there was awareness of bacterial endotoxins at the time, the primary focus was on their role major gastrointestinal events (food poisoning, enteritis etc.). In 1913 one of Metchnikoff’s primary associates in the Pasteur Institute, microbiologist Arcangelo Distaso, stated that in nervous disorders inclusive or diarrhea and/or constipation, the liberation of gram-negative bacterial endotoxins appeared to be at play. Distaso provided little clinical evidence to back up his assertion, other than the loss of viable *E. coli* in stool samples obtained from a few human cases of diarrhea and constipation. Distaso interpreted the loss of viability in these cases (and his experiments with dogs, monkeys, guinea pigs) as a sign of increased bacterial destruction and liberation of *E. coli* endotoxins within the gut [68]. Despite being ever so close to a rational path in commensal endotoxin research (viz mental health), over the next decade Distaso instead continued to focus his research attention on the products of intestinal microbial action on dietary proteins (indole, skatole and their conjugation sulphates) – proving them to be systemically available, and certainly a product of diet and intestinal microbial make-up, but not proving them to be harmful [69]. At the same time, in 1913, famed British physician Sir Frederick Andrewes, referring to intestinal toxemia, stated that ‘*Bacteria are, of course, constantly undergoing dissolution in the alimentary canal, and one cannot dispute the possibility of harmful effects from such endotoxins*’. However, he went on to lament the fact that the role of the endotoxin production by non-pathogenic intestinal flora in autointoxication symptoms had not been investigated with vigor. Overall, Andrewes doubted a role of commensal endotoxin production, with one somewhat prophetic exception – colon bacillus (*E. coli*) endotoxin production [70]. As we will discuss later, lipopolysaccharide endotoxin (LPS) production by commensal bacteria, particularly in the context of intestinal permeability, is emerging as an important factor in systemic health. Despite a century worth of investigations involving the effects of experimental endotoxin administration [71], it wouldn’t be until 2001, with the landmark study of Reichenberg, that the detrimental effects of low-level systemic LPS on mental and cognitive health would be appreciated [72].

In 1922, Boston physician Issac Jankelson described ‘chronic fermentative intestinal indigestion’ akin to modern day small intestinal bacterial overgrowth. Driven by fermentation of carbohydrates in the ileum, the condition was characterized by chronic diarrhea, bloating, depressive symptoms, fatigue and anxiety. Indeed Jankelson described the syndrome as a common antecedent to cases of neurasthenia (referred to by him and others as “enteros-thenia”) and often associated with an overgrowth of clostridium species [73]. In a series of experimental studies in the late 1920s, Loyola University physician and microbiologist Lloyd Arnold reported that small intestinal bacterial overgrowth and accompanying intestinal permeability are encouraged by environmental (e.g. heat) stress, the introduction of potentially pathogenic bacteria, nutritional deficiencies, and/or marked deviations in diet (e.g. transfer to a very high protein diet). Later he would demonstrate that the type of diet consumed by animals over one month could influence the detrimental effects, even mortality, when an intestinal pathogen was introduced via stomach tube. Arnold reported that dietary context can influence intestinal-pathogen-induced symptoms and mortality. Remarkably, he found that the standard stock rodent chow, one that they had been using for years in his lab to raise mice, was associated with the highest mortality in mice upon *Salmonella enteritides* exposure vs. other natural foods. In particular, a switch from the standard chow to banana powder decreased mortality from 96% to 6% over one month post-infection [74-76]. For Arnold, the food vehicle was an important factor in intestinal microbiology outcomes. In 1937, following up on the work of Arnold, Yale University microbiologists reported that banana, apple and raisin powder can significantly elevate the lactic acid microbiota in the intestine of animals [77]. Arnold was also one of the first physicians to ponder how the hunter-gatherer and ancient diets could influence intestinal microbiota, which in turn, could influence human survival [78].

In 1926, microbiologist Arthur Issac Kendall also reported alterations to the intestinal microbiota in those with intestinal intolerance for carbohydrates (the key symptom of which, in addition to alternating constipation/diarrhea, was “neurosis”), with a reduction in overall lactic acid bacteria. He theorized that the gas-producing bacteria were migrating upward and producing small intestinal bacterial overgrowth [79]. An additional consideration was the extent to which alterations in stomach acid production could influence intestinal microflora in small intestinal bacterial overgrowth and mental health disorders. In 1935 gastroenterologist Theodore Althausen reported low stomach acid in 2/3 of patients with carbohydrate intolerance (again reporting alternating constipation/diarrhea and anxiety as key symptoms) [80]. Both Kendall and Althausen reported clinical success with acidophilus milk as part of the treatment. In 114 cases of various mental disorders, physician W.M. Ford Robertson reported that 54% of patients (vs. 20% of healthy controls) were outside of normal gastric acid production, the majority being in a state of hypochlorhydria. Robertson concluded that the loss of the bactericidal effects of normal gastric acidity could have far reaching effects in mental health, including neurasthenia and in those “borderland patients” that otherwise presented with sub-threshold symptoms [81]. In 1912 Francis Brook, a physician at Guy’s Hospital in London, began to examine the fecal microflora of neurasthenic patients, reporting significant differences in about half of 132 patients encountered. In particular he reported differences in total coliforms and streptococcus group bacterium using culture technique [82]. Later, Geoffrey Shera, a physician at the East Sussex Mental Hospital, reported that among 53 newly admitted patients (within one week of arrival) there was significantly lower fecal *L. acidophilus* in 80% of patients and significantly higher Streptococcus spp. vs. several normal controls [83].

It was also during the intestinal toxemia era that researchers began examining the ways in which broad aspects of the diet – fiber-rich grains, fruit and vegetables, as well as protein-rich eggs, meat, milk – could influence the intestinal microflora. For example, in 1910 Herter and Kendall showed for the first time that diets dominated by protein could shift the bacterial microflora in monkeys and cats, increasing proteolytic bacteria and decreasing lactobacillus and bifidobacterium spp. The opposite was reported when the animals were placed on a carbohydrate diet inclusive of milk. Interestingly, Herter and Kendall also made note of behavioral changes in association with the intestinal microflora alterations, monkeys in particular were reported to experience lassitude, cognitive difficulties and general disinterest in environmental stimuli [84]. In the decade following this landmark investigation, others would also substantiate the influence of diet in the make-up of intestinal microflora.

Others began examining the ways in which animals without intestinal microflora, those raised in sterile conditions, differ from conventional animals. In 1912, Michel Cohendy of the Pasteur Institute not only successfully raised germ-free chickens, he reported them to be very resilient to various environmental stressors (e.g. hunger, thirst, cold, and other climate stress) [85]. The editors of the *Journal of the American Medical Association* (JAMA) heralded the news of Cohendy’s work by stating that ‘*It will henceforth be possible to infect an organism that is free of microorganisms and study the consequences…our attitude toward the microbial population inhabiting our digestive tube will in consequence assume a different significance*’ [86]. Sadly, it wouldn’t be until 2004, in a Yakult-funded study, that researchers would give attention to the brain and behavioral aspects of germ-free development and the consequences of introduction of strains of probiotic [87]. It is also noteworthy that during this time researchers identified various strains of lactobacilli, including Metchnikoff’s L. bulgaricus, in garden soil and plant foods [88]. These findings, as discussed in Part II, are of relevance to the modern investigations involving germ-free animals, stress physiology, and cumulative contact with non-pathogenic bacteria via food and incidental contact via time spent in nature (e.g. gardening, farming, nature-based recreation etc.).

Modern medical writers point to the 1958 study of Eiseman, et al. as the first report using fecal microbiota transplantation (fecal enema) with successful outcome [89]. These surgeons obtained feces from healthy donors, suspended it in saline, and transferred it to four patients with enterocolitis as a retention enema. However, it was during the autointoxication era, almost four decades earlier, that New York physician N. Philip Norman published papers on successful enema-based transfer of bacteria. Specifically, Norman would isolate lactobacilli and non-pathogenic microbes obtained from the stool of healthy infants, mix into a lactose solution, and inject the bacteria into the adult cecum via tube. For Norman, dissatisfied with the results of oral lactobacillus preparations alone, correcting a disordered intestinal microbiota via transfer of non-pathogenic intestinal organisms was central to the treatment of many chronic medical conditions [90,91]. He theorized that disordered gut microbiota set in motion a systemic ‘protective degeneration’ in that the normal defensive response to the chronic toxin load was the ultimate cause of cellular dysfunction. Psychological stress was a catalyst – in 1920 he wrote - ‘*one familiar with a neurology based on an evolutional conception cannot fail to understand that psychic stress is capable of upsetting metabolic or endocrine harmonies’*[90]. A separate group of physicians from New York employed a similar technique, injecting freshly prepared L. acidophilus cultures via a colonic irrigation tube and reporting it to be successful as an adjuvant in arthritis care [92]. Meanwhile, long-time editor of the *American Journal of Gastroenterology*, New York physician Anthony Bassler, claimed clinical success with rectal infusions of cultured non-pathogenic commensal bacterium obtained from patients and other adults. Although Bassler claimed that ‘*it is probable that future years will show that many of the diseases classed today as of obscure origin will be understood to be directly or indirectly due to states of chronic toxemia from the intestinal canal*’, he found little value in specific species of oral lactic acid bacteria. He felt the legitimate paths of research in intestinal toxemia were being obscured – ‘*to jump to conclusions from a clinical case to the use of a single organism as the cure of them all puts just opprobrium on it, for such hit or miss medicine makes for commercialism, and inhibits the attention and work of the best workers in medicine…this slipshod therapy is the cause of advertising and lay institutions engaging in it*’ [93]. Bassler did not see non-pathogenic fecal coliform commensals as the autointoxication enemy; rather, after a decade worth of use, he reported them to reset a normal intestinal microflora and provide clinical value when instilled in live viable form via a rectal enema [94].

One of the more intriguing interventions in the treatment of nervous disorders connected to intestinal toxemia was the use of so-called colon vaccines. These were pioneered by New York physician George R. Satterlee, and as he reported in JAMA, his technique was to isolate coliforms from fecal material and deliver the vaccine (10–25 million heat-killed bacteria) subcutaneously. Providing the vaccine, once a week with incrementally higher doses up to 300 million bacterial units over three months, it was claimed that the intervention was associated with marked improvement in almost all cases [95]. He reported an initial exacerbation of symptoms and local irritation for 24–72 hours, followed by a marked and ‘decided relief’ of the baseline symptoms. In over 500 cases of depression and/or anxious states, Satterlee concluded that ‘*disturbances of the gastro-intestinal system are more often the cause of nervous symptomatology than the result of a diseased nervous system’*. The clinical gauge of success with colon vaccine was dependent upon, according to Satterlee, ‘*above all, the mental improvement*’ [96]. Although Satterlee wrote extensively on the use of his vaccines, there is no indication that the technique was adopted by a large number of physicians and as a result, his claims remained without validation. Gastroenterologist Bassler was one of the few who adopted the technique – heat-killed fecal coliforms for subcutaneous use, and live viable commensal bacteria for rectal instillations, with benefits noted particularly in cases of chronic fatigue and neuroses [93,94]. However, one of the leading pathologists of the time, Sir Philip N. Panton dismissed most of it as placebo – writing on vaccines for oral and intestinal toxemia he stated, ‘*nothing is more simple than the preparation of such a vaccine for any and every condition, and nothing would be more futile were it not for the mental effect of a hypodermic injection upon a confiding patient*’ [97].

In 1945, a group of researchers from Denmark produced a report in English entitled ‘Senility and intestinal flora: a reexamination of Metchnikoff’s hypothesis’. The study involved detailed fecal analysis of 63 older adults (aged >70) living in an institute for the aged. In 100% of the young healthy control group they found bifidobacterium in numbers ≥10^8^ per gram of feces, while only 44% of older adults without dementia met the same level of ≥10^8^ of bifidobacterium per gram of feces. Most striking was that only 9% of the older adults with dementia had bifidobacterium in numbers ≥10^8^ per gram of feces, and that these same older adults with dementia had the highest level of clostridia species [98]. The report received little international attention or follow-up; as discussed below, by this time even the popular media were dismissing autointoxication and a gut microbiota-brain connection as nonsense.

#### The sun sets on autointoxication and acidophilus therapy

“*Only the more senior members of the specialty would remember the excitement which was caused in the mental hospital world by the researches of Metchnikoff, and the statements concerning the Bacillus Bulgaricus. At the time there were no laboratories* [to make Bulgaricus sour milk] *at Rainhill Asylum and many other places, though there was a good one at Wakefield. Dr Wiglesworth, who was the speaker’s chief, was greatly interested in the subject, and he gave many of the patients plenty of sour milk. But when subjected to what might be called the “acid test” they did not, after some months of treatment, show any difference mentally*.”

W.F. Menzies, M.D. - *British Journal of Psychiatry*, 1930 [99]

Since autointoxication was a catch-all classification, connected to virtually every acute and chronic medical condition imaginable, even at its zenith it was still viewed with a degree of skepticism. Even those who supported the notion of intestinal microbial involvement in systemic disease conceded that the term made no rational scientific sense, at least not in the context in which it was used. There was little ‘auto’ (self; spontaneous) in intestinal toxemia - autointoxication was a more apt descriptive for the spontaneous generation of toxins by human cells – not a descriptive for the activities and consequences of microbial inhabitants. Writing in the *British Journal of Medicine* (1912), famed pediatric surgeon Hastings Gilford stated that ‘*autointoxication, the term as we commonly use it is significant of nothing but a sort of mental flatulency on the part of the user as a substitute for thought*’ [100]. The voices of its opponents, those who rightly pointed out that treating intestinal toxemia was practicing medicine by hypothesis alone, became increasingly louder. By the early 1920s several studies would take the gloss off the medico-scientific appeal of autointoxication, intestinal toxemia, focal infections, and their connections to mental health disorders. The once appealing urine test of indican (suggesting putrefactive tryptophan breakdown by microbes) was proving itself to be of little clinical value – perfectly healthy adults were demonstrating high amounts of urinary indican, while those with significant constipation and/or behavioral complaints often had low urinary indican levels [101].

Despite the enthusiasm related to a theoretical clinical value of lactic acid bacteria potions and milk products in those with mental health disorders, the actual benefits of administration were not realized. In three small published case series, acidophilus milk was reported to improve constipation and diarrhea, but not behavioral or mental status of patients, at least not in those with more serious forms of mental illness such as schizophrenia [102-104]. Moreover, researchers from New York were unable to detect differences in the presence or absence of *L. acidophilus* in the stool of 187 patients with a variety of mental health disorders vs. controls (although they did not quantify the *L. acidophilus* levels, reporting merely whether or not it was present at all) [105].

There was also a growing discontent among physicians concerning the marketing of bacterial products within medical journals and beyond. Autointoxication, by being both scientifically vague and broad in its potential application, became a Petri dish for the growth of quackery and charlatanism - so-called colon-cleaning products, dubious “colonic” contraptions and unsafe, lay-administered protocols stood side-by-side with largely unregulated bacteriotherapy products. Purveyors of what would later become known as probiotics were often part of fantastical claims built upon questionable products. Even prior to the 1927 study by Lawrence H. James in JAMA showing that only 13 of 107 commercial *L. acidophilus* or *L. bulgaricus* preparations had correct labeling and sufficient viability [106], there were concerns about the marketing over-reach. An editorial in JAMA [107] took exception to Intesti-Fermin advertisements placed in the *New York Medical Journal* wherein the company (Berlin Labs) sought physician alliance – the advertisement header stated “*We are telling the layman about Intest-Fermin…may we count on your assistance in spreading this message to everyone*?” In the emerging world of evidence-based practice, the short answer was no, Berlin Labs could not count on physicians to spread the word about an agent lacking scientific support. Put simply, bacterial supplements were increasingly being viewed in the same category as pseudo-scientific patent medicine and nostrum cures. By the mid-1930s even the dairy industry was distancing acidophilus milk from medicinal properties. In a 1938 article [108] entitled ‘Lactobacillus acidophilus milk gets psychoanalyzed’, one indicating a more than subtle shift toward Freud, dairy scientist Theron H. Butterworth suggested that the time had come to ‘*merchandise acidophilus milk as a superior fermented milk beverage – not as a medicine*’. Whether it was in heed to Dr Butterworth’s advice or not, the mainstream advertizing of acidophilus and autointoxication in tandem largely disappeared.

#### Autointoxication ‘debunked’ by Alvarez and Donaldson?

Walter C. Alvarez, an internal medicine physician at the University of California, San Francisco, was one of the most vehement critics of the autointoxication theory, at least as it pertained to chronic functional constipation. Although modern historians also equate autointoxication exclusively with constipation, there are multiple physician reports during the autointoxication era claiming that diarrhea and/or alternating constipation and diarrhea was equally, if not more often, a key symptom of neurasthenia/melancholia due to autointoxication. New York physician William H. King is an example among many; he wrote in 1913 that among 300 of his reported cases, ‘*alternate constipation and diarrhea is a far more diagnostic symptom*’ and that this was, in turn, tied to greater frequency of functional nervous symptoms [109]. Alvarez focused on constipation, stating that autointoxication was merely being used as a diagnostic ‘*cloak of ignorance*’ for mental illness or legitimate underlying organic disease states. Alvarez challenged the experimental studies showing the harmful effects of indole (and most of the other putrefactive substances) in animals, stating that they were of little relevance to humans. He presented research challenging the notion that putrefactive substances could be absorbed from the intestinal tract in anything other than trace amounts, and he also criticized the microbial culture techniques as used in studies supportive of Metchnikoff [110,111]. Alvarez wrote, ‘*any scientifically trained man who would read Metchnikoff’s book on the Prolongation of Life will probably stop to wonder how a man in his position could have written so positively on the basis of so little exact proof*’ [111]. Alvarez reported anecdotally that he could provoke classical symptoms of autointoxication with masses of barium and cacao butter suppositories. He was of the opinion that in chronic constipation, lower bowel distention sets in motion a “reverse peristalsis”, a retrograde flow of intestinal contents back toward the stomach [110]. This backward mechanically-induced movement, or as he called it, ripples coming up the tract (enough to bring colonic materials to the tongue), brought about many of the symptoms of autointoxication – it was very common, he opined, in ‘*nervous, worn-out women*’. Even more specifically, he stated that ‘*some of the most striking manifestations of reverse peristalsis are observed in the hysterical*’ [112]. It was simply mechanical forces acting upon these highly sensitive, nervous individuals, and informing them of their mental involvement would, as he claimed, ‘s*et them free*’. It is, of course, debatable whether or not informing a so-called nervous, worn-out woman that she is responsible for colonic material appearing on her tongue, would indeed, set her free. There were many who rallied to support Alvarez; the response by Edward Goodman, a University of Pennsylvania gastroenterologist, clearly highlights the chasm and lack of middle ground related to intestinal toxemia – ‘*The suggestion that symptoms are due to mechanical distention and irritation may not meet with the approbation of the army of toxemiaphils but will certainly be a welcome suggestion for us in the anti-toxemia squad*’ [113]. Among the toxemiaphils writing to JAMA to criticize Alvarez, there was New York physician William Howard Hay. He held firm that in constipation, an acceleration of food residue transit through the colon was associated with better health in conjunction with urinary indican reduction [114]; he also, it should be pointed out, maintained that autointoxication was responsible for *all* human ailments, including baldness. These vocal armies and squads were in opposition; there were few considering the possibility of mechanical distention, psychological inputs, and, for example, intestinal permeability and gut-derived endotoxins working in concert and in a bi-directional fashion.

In 1922, Arthur N. Donaldson used petroleum-soaked and barium-coated cotton pledgets packed into the rectum of four otherwise healthy volunteers, reporting that after three hours the procedure could provoke the classic mental symptoms of intestinal toxemia. Since the rectal plugging for three hours subsequently brought about neuromuscular fatigue and prolonged mental reaction time, it was presumed that the symptoms of autointoxication could be explained by mechanical distention. Donaldson also had 5 adults in the same case series volunteer in refraining from any bowel movements for 90 hours. This process induced mental symptoms of depression, irritability, loss of attention and headache. One hour after the first permitted bowel movements (most used an enema), Donaldson queried the subjects and they reported the mental symptoms to be alleviated (except in one subject where the headache was alleviated 3 hours after the bowel movement) [115]. Donaldson, erroneously it would turn out later [116], assumed that it would take a significant amount of time for putrefactive amines to be removed from the blood; therefore, in his opinion, a relatively rapid improvement in symptoms could only infer mechanical causes (distention) as the root of autointoxication. Alvarez made the same assumption, stating in his oft-cited paper ‘*one does not sober a drunken man immediately by taking his flask of whiskey away from him*’ [111]. As we will discuss at length in Part II, intestinal microbe-derived lipopolysaccharide endotoxin (LPS) is not alcohol; in healthy adults without abnormal intestinal permeability, LPS is rapidly cleared by the liver after systemic absorption (just as uremic toxins are cleared rapidly by the healthy kidney) [117]. However, a very low level of a single dose of intravenous LPS has neurobehavioral consequences [118]. Low level LPS can influence mental outlook, headache and cognition, and yet these neurobehavioral deficits are typically past peak within two hours [119,120]. Alvarez and Donaldson showed that mechanical distention can cause general discomfort, headache and cognitive changes; modern evidence certainly supports their findings. However, their work no more disproved the central tenets of intestinal toxemia – i.e. that intestinal microbes and/or microbial breakdown products can influence systemic health - than did Norman’s reports of success with intestinal microbial transplants prove them to be legitimate.

Despite this, modern medical historians, at least through the late 1990s, consistently point to Alvarez and Donaldson as providing the clear evidence that debunked autointoxication as nonsense [8-11]. It seems the more time passed, the more monumental and iron clad this work was perceived to be. A modern myth concerning Alvarez and Donaldson, one that keeps spinning, is that which states their research was both rigorous and decisive – it was neither. Accepting, for a moment, the wrongful assumption that intestinal toxemia was the exclusive realm of chronic constipation, Donaldson, for all intents and purposes, had attempted to set up a 90 hour model of functional constipation in 5 healthy adults. The massive caveat is, of course, that they were *healthy* adults *suppressing* a normal urge to defecate; they were not adults with chronic illness (e.g. major depressive disorder) and co-morbid functional constipation. The extent to which holding back bowel movements for 90 hours was itself a psychological stressor, one capable of provoking symptoms in these 5 healthy adults, was never discussed by Donaldson or Alvarez. What we know from contemporary evidence is that in much larger studies of functional constipation, the fecal concentrations of *Lactobacillus* and *Bifidobacterium* are significantly lower and intestinal permeability is significantly higher vs. healthy adults without constipation. Furthermore, in functional constipation there is an enhanced systemic immune response, almost certainly due to larger molecules gaining access across the intestinal barrier [121]. How does this fit in with the overlap between depression and constipation [122], and the more specific finding of longer whole gut transit time positively correlated with depression [123]? Undoubtedly, constipation may be a mere surrogate marker for other risk factors – lack of exercise, Western diet, obesity, etc. - in depression, type II diabetes, and other chronic diseases [124]. However, the question remains - why are the more severe forms of non-pathological chronic constipation associated with cardiovascular disease risk even when other lifestyle variables (diet, medications, exercise, etc.) are adjusted [125]? Intestinal permeability may be at play.

Recto-gastric and recto-colonic reflexes, manifest through mechanical distention, are now active areas of research in IBS and functional intestinal disorders. Indeed, recent studies involving experimental rectal distention have shown the induction of postprandial nausea and upper gut symptoms along with delayed gastric emptying and altered colonic transit in humans [126-128]. Alvarez and Donaldson were certainly correct in that regard. Undoubtedly, stressful life events have also been associated with an increased frequency of functional constipation [129]; however, this does not remove microbes from playing an important role in the modulation of gut motility and any potential systemic health consequences of chronic constipation. As discussed later, methane-producing intestinal microbes have been linked with constipation and are now emerging as a key part of reflex contraction and altered motility in IBS [130-133]. As we will discuss in Part III, beneficial microbes have recently been shown to reduce physio-behavioral signs associated with colorectal distention.

#### Alvarez – migraines and ulcers

As for Alvarez, he was not a man to shy away from opinion. He wrote that ‘*in the worst of these cases of “autointoxication” the sufferers are undoubtedly psychopathic. I have learned to recognize the type at the first interview, and I no longer waste much time with them, as it is practically impossible to change their habits of thought. They are generally of neurotic or insane ancestry and often give a history of “nervous breakdowns” in the past’*[110]. Alvarez, himself, appeared to fall victim to the next wave of popular medical culture between the 1930s through the 1960s – seeing the psychosomatic at every clinical turn. Embracing a sort of whole-body phrenology, Alvarez wrote of migraine patients, ‘*In 95 percent of the hundreds of my women patients, I found social attractiveness, a better-than-average intelligence, and a decided quickness of thought and bodily movement. Three out of four of the women had a small, trim feminine body with well-formed breasts*’ [134]. He went on to urge physicians to be on the lookout for attractive women with this body-type, and to query these female patients about migraine. Writing on the curative properties of antibiotics and their ability to extend life among those with mental disease, he stated that one in eight men ‘*is either not intelligent enough or not sane enough to work, even in a labor battalion. Unfortunately, too, these people breed their kind day after day’.* Going on to state that *‘It is important to face the fact that today antibiotics are keeping alive hundreds of thousands of persons who are a drain on the community’,* and concluding that *‘modern medicine is keeping alive those who will never pull their weight in the boat*’ [135]. Calling out such statements from Alvarez is not mere digression. In the revisionist historical view of Alvarez as a man of science, one who, at least according to the contemporary reviews, almost singlehandedly debunked ‘autointoxication’, it is important to recognize that he developed completely unscientific and controversial views on neurological and mental health conditions. There is no question that his own views influenced his research interests; as disclosed in his personal autobiography (pg. 58) Alvarez reported that his own many experiences of immediate relief from abdominal distress and headache after a bowel movement is what led him to investigate the effects of packing the lower bowel with the cotton – ‘*Whenever I have been constipated, and these symptoms have distressed me, they have stopped the instant the constipation was overcome. Eventually, I gathered and wrote up so much evidence against the idea of “autointoxication” that most doctors stopped making this diagnosis*’ [136]. Moreover, he describes (pg. 59) his research focus was, as of 1912, studying the top-down influence of a disturbed mind on the body, rather than a disturbed body on the mind [136].

He had little confidence in the infectious theory of peptic ulcers, and described the individuals with them as the “*go-getter*” type – ‘*he is often a keen, wide-awake, sensitive type of man who drives himself all day and part of the night, who responds too much to things that break into his routine, and does not know how to relax*’ [137]. If there were any doubting his position, Alvarez addressed the Annual Meeting of the American College of Physicians (April, 1932) on the topic of ulcers and stated that in many cases what the patient needs is “*to take a little vacation now and then when the stomach gets to hurting*” [138]. For decades he held on tightly to his belief that ‘*commonly, the biggest factor in the production of an ulcer is a psychic one*’ [139]. In the end, the psychosomatic convictions of Alvarez and the so-called go-getter personality were deemed to be of minor relevance in ulcer; it was the gastric pathogen *Helicobacter pylori* at work, a microbe that appears to be mostly, if not completely, unimpressed if the patient takes a little vacation or not [140]. This, too, is not mere digression. *H. pylori* infection has recently been linked with extragastric diseases including dementia, depression, cardiovascular disease and type II diabetes [141-144]. Recent studies also show that animals infected with *H. pylori* subsequently display delayed gastric emptying and visceral hypersensitivity [145] - alterations that Alvarez considered to be top-down and psychosomatically driven. On a final note related to migraine, it is also telling that recent studies have associated *H. pylori* with migraine, and eradication with improvement in symptoms [146,147]; this, regardless of whether the female patients had a small, trim and so-called feminine body.

#### Hepatic encephalopathy and gut microbes

In the mid-1960s, with the recognition that antibiotics and *L. acidophilus* can help reduce the systemic ammonia load related to the symptoms of hepatic encephalopathy, there were some renewed and fleeting references to intestinal autointoxication [148]. Earlier, in 1924, a small study by researchers from the New York State Psychiatric Institute reported that, compared to sterile milk, *L. acidophilus* milk reduced seizure frequency among 12 patients during a 3-month administration period [149]. However, hepatic encephalopathy was and continues to be viewed as a condition primarily involving the dysfunction of hepatocytes and/or hepatic blood supply – in the clinical realm there was no extension of thought suggesting that antibiotic/probiotic utility in hepatic encephalopathy represents a gateway to the clinical treatment of mental health disorders. In other words, the treatment of hepatic encephalopathy by manipulation of the intestinal microbiota (and therefore blood-to-brain toxin burden) in the 1960s was not a validation of intestinal autointoxication, nor was it the birth of the modern gut-brain-microbiome axis – it was of little relevance to clinicians attending to patients with depression, an unresolved oedipal complex, and normal basic liver function tests. Those were the halcyon days of Freudian psychodynamics, when depression and suicide from high places were literally being explained away, in mainstream medical journals, as oedipal guilt in concert with phallic symbols [150], where chronic constipation was described as ‘*a self-evident case of hypochondriasis with an undoubted ano-erotic hysterical element*’ [151,152]; we had fully arrived at a place where theory once again permeated clinical interpretations and interventions.

#### Autointoxication baton passed to Freud

As intestinal toxemia faded away, and Freudian psycho-neuroses and top-down psychosomatic theories became popularized as a means to explain so-called ‘functional’ disorders, Alvarez’s view would linger for decades. Indeed as late as 1976 one of the leading gastroenterology textbooks would state that patients with reported carbohydrate intolerance and symptoms in line with autointoxication were ‘*essentially unhappy individuals…any suggested panacea and therapeutic straw is grasped. No regime is too severe, and no program too forbidding. With the tenacity of the faithful they grope their way from one physician to the next in a relentless search for a permanently successful remedy’*[153]. To fully appreciate how far the pendulum had swung, consider that in 1989 physicians writing in the *Journal of Clinical Gastroenterology* (JCN) argued that lingering notions of concern related to intestinal autointoxication among patients (and a small minority of doctors) are simply explained by Freudian psychology [11]. Specifically, they are a result, postulated these doctors writing in one of the premier gastroenterology journals, of conflicts between child and parent over control of defecation during toilet training. They floated this bold hypothesis after informing the reader that Alvarez, Donaldson, and their cohorts had adopted rigorous approaches and refined techniques - ‘*advances invalidated the medical credibility of intestinal autointoxication*’. Of course, many of the suppositions of intestinal autointoxication were never scientifically invalidated; they were simply never thoroughly examined at all. The core theoretical tenet bears repeating – i.e. that intestinal microbes, directly and/or indirectly, could influence systemic health, including that in the mental realm. This was certainly not proven, but it was not even remotely invalidated. Still, there were no letters to the editor of the JCN in 1989 to challenge a Freudian explanation, peer-reviewers evidently found the premise acceptable enough to publish. And so it was that legacy of autointoxication was deemed to be that of complete nonsense; a by-gone era of medical malpractice (high-risk surgery based on theory) and the commercial spin of shysters who co-opted and diminished the legitimacy of fledgling discoveries.

#### Taboo to topical

Ultimately, these forces, along with absolute lack of human evidence, would block rational lines of potential investigation (e.g. intestinal permeability, LPS endotoxemia, proper investigations of select probiotic strains) and allow for Freudian theory – toilet training conflicts - to provide a default answer that could only be described as extravagant, convenient, and scientifically unverifiable. Only a few in primary medicine dared raise the spectre of Freud as pseudo-scientist [154]. No one, until 2003, dared raise the suggestion in a scientific journal that the administration of beneficial microbes might have an adjunctive place for the treatment of human fatigue and depression, after all. However, a 2012 review in a leading psychiatry journal, referring to the hypothesis of our group, stated the following ‘*Logan and Katzman first proposed the use of probiotics as adjunct therapy in the management of depression. Since then there has been an accumulation of data from both clinical and preclinical studies supporting the view that probiotics may have a role in the treatment of depression*’ [4]. Setting aside that the first proposed use of probiotics for depression was made a century prior, by Scottish physician Hubert J. Norman [155], the larger question is how did we get here? How did a taboo subject such as microbes *for* mental health, one banished for over 70 years, insinuate itself back into to mainstream psychiatric thinking in just under a decade? The rapid advances that justify some bridled enthusiasm and further outside-the-box thinking, in the context of translational medicine, will be discussed in Parts II and III.

## Competing interests

ACB and EMS have no competing interests. ACL has received consulting fees from Genuine Health, Toronto, Canada.

## Authors’ contributions

ACB, ACL and EMS contributed equal time and effort in the investigation, research and drafting of this manuscript. All authors read and approved the final manuscript.
